# Relationship between symptoms of dry eye syndrome and occupational characteristics: the Korean National Health and Nutrition Examination Survey 2010–2012

**DOI:** 10.1186/s12886-015-0147-3

**Published:** 2015-10-29

**Authors:** June-Hee Lee, Wanhyung Lee, Jin-Ha Yoon, Hongdeok Seok, Jaehoon Roh, Jong-Uk Won

**Affiliations:** 1 Graduate School of Public Health, Yonsei University, Seoul, Korea; 2 The Institute for Occupational Health, Yonsei University College of Medicine, Seoul, Korea; 3 Department of Preventive Medicine and Public Health, Yonsei University College of Medicine, 50 Seongsanno (134 Sinchon-dong), Seodaemun-gu, Seoul Korea

**Keywords:** Occupational category, Symptoms of dry eye syndrome, KNHANES

## Abstract

**Background:**

Dry Eye Syndrome (DES) is a broad spectrum of uncomfortable ocular conditions that are caused by reduced production of tears or an increased tear evaporation rate. This study evaluated the relationship between symptoms of DES and occupational characteristics to identify the occupation-dependent differences in the prevalence of symptoms of DES using the Korean National Health and Nutrition Examination Survey V (2010–2012) data.

**Methods:**

A total of 6023 participants were included (3203 men and 2820 women). Questionnaires and physical examinations were used to record clinical characteristics, occupational characteristics and medical history. Odds ratios (ORs) and 95 % confidence intervals (95 % CIs) for symptoms of DES were calculated according to the occupational characteristics.

**Results:**

Among the participants, 963 persons (16.0 %) had symptoms of DES. An increased risk (relative to the green-collar group) was observed for the ordinary white-collar (OR, 1.73; 95 % CI, 1.73–1.41), executive white-collar (OR, 1.40; 95 % CI, 1.02–1.92) and skilled blue-collar (OR, 1.44; 95 % CI, 1.04–2.00) groups. Furthermore, paid workers had a significantly higher risk of dry eye symptoms (OR, 1.21; 95 % CI, 1.02–1.45), compared to self-employed workers.

**Conclusion:**

Our study is the first research to reveal that white-collar workers have a higher risk of symptoms of DES than blue-collar workers, that skilled blue-collar workers have a higher risk than unskilled blue-collar workers, and that paid workers have a higher risk than self-employed workers.

## Background

Dry eye syndrome (DES) is one of the most common complaints worldwide among patients who visit ophthalmic clinics [1]. DES is a commonly used clinical term that covers a broad spectrum of ocular conditions that are characterized by irritation and discomfort on the eye surface, due to reduced production of tears or an increased tear evaporation rate. Its diagnosis is mainly based on subjective patient-reported symptoms, and therefore it is reported as all cases that involve complaints of subjectively perceived symptoms, without an objective clinical diagnosis [2, 3]. Common symptoms of DES include dry eyes, a foreign body or a burning sensation in the eyes that is accompanied by excessive tearing and light sensitivity (photophobia) [4]. In severe cases, discomfort can persist or eye surface injury may occur [2].

The pathogenic mechanism of DES begins with a circulatory disorder of tears, due to a disorder in the lacrimal gland, and results in subsequent tear film instability. This leads to a lesion on the surface epithelium, which then leads to decreased tear production in the tear gland, subsequently aggravating the DES symptoms, and ultimately inducing an inflammatory response [5, 6]. The typical risk factors for DES are old age, female sex, smoking, use of contact lenses, refractive surgery and living in a dry environment [7–9]. There are also reports that DES is significantly associated with both ocular discomfort and psychological states, such as depression and anger, and that it adversely affects the patient’s quality of life [10, 11]. Therefore, DES is no longer a simple pathological state that is limited to ocular disorders, as it has become a broader issue that affects quality of life [12].

The major inclusion criterion that was used in previous studies to assign subjects to the DES group was an affirmative response to the DES-specific symptoms listed in a self-reported questionnaire. One study used self-reported DES symptoms, and reported that the prevalence of DES was higher among adult women than that among adult men (17.9 % vs. 10.5 %); this prevalence was observed to increase with age [13]. Other studies have reported widely differing prevalences (5–30 %) among adults who were ≥50 years old [14–16]. In South Korea, a DES prevalence of 14.4 % in the general population was derived from DES-related data in the Korean National Health and Nutrition Examination Survey (KNHANES) (2010–2011) [17]. However, while researchers have extensively studied the age-related prevalence and general factors for DES, few studies have investigated the occupational characteristics of DES.

Interestingly, one study that investigated DES-related occupational characteristics reported that DES was positively related to the frequency of eye blinking, and that a prolonged duration of working at a video display terminal increased the prevalence of DES [18]. As DES can affect quality of life and psychological status, thus potentially limiting an individual’s productivity, it is important to identify high-risk occupational groups [10, 11]. To address this issue, the present study aimed to evaluate the relationship between symptoms of DES and occupational characteristics, thus identifying the occupation-dependent differences in the prevalence of symptoms of DES. Using KNHANES data, which is representative of the health and nutritional conditions of all South Koreans, occupational categories with a high risk of symptoms of DES can then be identified and investigated to reveal data for establishing basic strategies to prevent DES [19].

## Methods

### Subjects

KNHANES is a cross-sectional, population-based and nationally representative survey of the health and nutritional status of Korean civilians, and is managed by the Korean Centers for Disease Control and Prevention (KCDC) [20]. The participants are chosen using proportional systematic sampling with multistage stratification based on sex, geographical area and age-groups via household registries [20]. Trained interviewers administer questionnaires regarding the participants’ demographic, socioeconomic, dietary and medical history, and examiners then perform a physical examination either at the participant’s home or at mobile examination centers. All participants provided their written informed consent before participating in KNHANES. And then the present study’s design was approved by the institutional review board (IRB) of Korean Centers for Disease Control and Prevention (IRB: 2010-02CON-21-C, 2011-02CON-06-C, 2012-01EXP-01-2C).

**Fig. 1:**
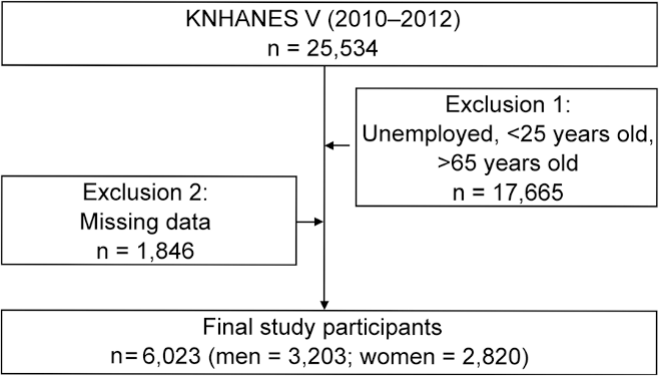
Participant data analysis

For this study, we obtained 3 years of survey data from KNHANES V (2010–2012), which included 25,534 individuals. To evaluate the relationship between DES and the participants’ occupational characteristics, data from unemployed individuals (e.g., housewives and military servicemen) and individuals who were <25 years old or >65 years old were excluded. Furthermore, participants with missing data regarding their physical examination or answers to the questionnaires were excluded. After these exclusions, data from 6023 participants (men, 3203; women, 2820) were analyzed (Fig. 1).

### Main variables

DES was defined using a self-questionnaire by answer for DES symptoms. Asking about associated DES symptoms is among the most reliable diagnostic and clinical approaching methods [2, 21], especially since there is no consensus of clinical diagnostic criteria for determining individuals with DES. Therefore, to investigate the prevalence of DES symptoms, subjects were asked the following question: “Until now, have you ever had symptoms of DES before: for example, a sense of irritation or dryness of the eye?” Possible answers were “yes” or “no”. Participants who had symptoms of DES were defined as the DES group, and others were defined as the non-DES group.

Next, we defined the participants’ socioeconomic status (education, household income and residence). Levels of education were defined as middle school, high school and university. The household income was estimated using standardization methods for classifications of sex and 5-year age groups, and compared to the standard income level for South Korea. The adjusted family income was then used to categorize the household income into quartiles. The residence (urban or rural) was classified primarily via population size, using the Korean administrative units, which define urban areas as having a population of >50,000 people [22]. The degree of obesity was evaluated using the participants’ body mass index (BMI, kg/m^2^), according to the Korean Society for the Study of Obesity criteria [23].

A self-administered questionnaire was used to investigate health behavioral factors such as a history of smoking, alcohol consumption (never, moderate, severe) and physical activity (no, low-intensity, high-intensity). Smoking history was categorized as never (<100 cigarettes in lifetime), former (had smoked in the past, but no longer smoked) or current smokers (a history smoking at the survey). Heavy alcohol consumption was defined as at least 7 alcoholic beverages twice or more per week for men, or at least 5 alcoholic beverages twice or more per week for women. Physical activity was defined as no activity, low-intensity activity and high-intensity physical activity (>20 min of strenuous activity more than 3 times per week).

Occupational categories were defined as executive white-collar, ordinary white-collar, pink-collar, green-collar, skilled blue-collar and unskilled blue-collar workers, based on the International Standard Classification of Occupations [24]. Executive white-collar workers included legislators, senior officials, managers and professionals. Ordinary white-collar workers included technicians and associated professionals. Pink-collar workers were clerks, sales persons or customer service workers. Green-collar workers were workers who were employed in agriculture, fishery or forestry. Skilled blue-collar workers included crafts persons, plant and machinery operators or assemblers. Unskilled blue-collar workers were defined as elementary workers.

Work type, work duration (h/week, categorized using the median value), work status (paid worker, self-employed worker, unpaid family worker), daily mean sunlight exposure and exposure to secondhand smoke at an indoor working place were defined using the health interview and health examination survey results from KNHANES V.

### Statistical analysis

The data were analyzed using SAS software (version 9.3, SAS Institute Inc., Cary, NC). Chi-squared tests with weighted analysis were used to compare differences in the baseline characteristics according to DES symptoms. In this study, 2 different logistic regression models were used to assess the relationship between occupational characteristics and DES symptoms: Model I was adjusted for age, sex and BMI, while Model II was adjusted for, age, sex, BMI, socioeconomic status (education, household income, and residence) and health behavioral factors (alcohol drinking, smoking and physical activity). Differences with a *P*-value of <0.05 in the two-tailed analyses were considered to be statistically significant.

## Results

In the study population (*n* = 6023), the weighted distribution according to sex was 51.88 % (*n* = 3203) for men and 48.12 % (*n* = 2820) for women. The weighted distribution according to age was 18.37 % (*n* = 1078) for participants who were 25–34 years old, 26.63 % (*n* = 1693) for 35–44 years old, 29.33 % (*n* = 1707) for 45–54 years old, and 25.66 % (*n* = 1446) for 55–65 years old. Urban residents (78.86 %; *n* = 4824) outnumbered rural area residents (21.14 %; *n* = 1446). The distribution according to household income was 2081 participants (31.85 %) in the 4th quartile, 1943 (31.68 %) in the 3rd quartile, 1520 (27.45 %) in the 2nd quartile, and 479 (9.02 %) in the 1st quartile. Furthermore, 2311 persons (33.79 %) were university graduates, 2160 (37.47 %) were high school graduates, and 1552 (28.74 %) were middle school graduates. The degree of obesity categories included obese participants (*n* = 2053; 34.59 %), overweight participants (*n* = 1484; 24.21 %), and normal weight participants (*n* = 2486; 41.20 %). Regarding smoking status, the majority of participants were non-smokers (*n* = 4385; 71.82 %), including 1330 former smokers; smokers accounted for 28.18 % (*n* = 1638) of the study population. Regarding the exposure to second-hand smoke, 47.40 % (*n* = 2795) of participants stated that they were exposed to it, including 11.94 % (*n* = 660) who stated that they were exposed for >1 h/day to second-hand smoke. Regarding alcohol consumption, 19.23 % (*n* = 1152) of participants were nondrinkers, and among participants who drank alcohol (*n* = 4871; 80.76 %). Among participants who drank alcohol 15.63 % (*n* = 914) were heavy drinkers and 65.13 % (*n* = 914) were moderate drinkers. The majority of the participants were physically no exercise group (*n* = 3846; 64.47 %), while the rest regularly engaged in low-intensity exercise group (*n* = 1871; 30.49 %) or high-intensity exercise group (*n* = 306; 5.04 %). The occupational categories included executive white-collar workers (*n* = 1410; 21.09 %), ordinary white-collar workers (*n* = 894; 13.30 %), pink-collar workers (*n* = 1326; 23.51 %), green-collar (*n* = 541; 9.63 %), skilled blue-collar workers (*n* = 1030; 17.54 %), and unskilled blue-collar workers (*n* = 822; 14.93 %). The majority of participants were daytime workers (*n* = 4942; 81.88 %), while evening or night time and night-shift workers accounted for 11.10 % (*n* = 654) and 7.02 % (*n* = 427) of the study population, respectively. The majority of participants worked <43 h/week (*n* = 3081; 51.44 %), while 48.56 % of participants (*n* = 2942) worked ≥43 h/week. Exposure to sunlight was <2 h/day for 63.60 % (*n* = 3942) of participants, 2–5 h/day for 22.94 % (*n* = 1325) of participants, and ≥5 h/day for 13.47 % (*n* = 756) of participants (Table 1).

**Table 1:** Basic characteristics of the study population

	Total (*N* = 6023)		
	No.	(%^a^)	%^b^
Sex			
Men	3203	(53.18)	51.88
Women	2820	(46.82)	48.12
Age, years			
25–34	1078	(18.20)	18.37
35–44	1693	(28.58)	26.63
45–54	1707	(28.81)	29.33
55–65	1446	(24.41)	25.66
Residence			
Urban	4824	(80.09)	78.86
Rural	1199	(19.91)	21.14
Household income			
1^st^ quartile	479	(7.95)	9.02
2^nd^ quartile	1520	(25.24)	27.45
3^rd^ quartile	1943	(32.26)	31.68
4^th^ quartile	2081	(34.55)	31.85
Education level			
Middle school	1552	(25.77)	28.74
High school	2160	(35.86)	37.47
University	2311	(38.37)	33.79
Degree of obesity			
Normal	2486	(41.28)	41.20
Overweight	1484	(24.64)	24.21
Obese	2053	(34.09)	34.59
Smoking			
Never	3055	(50.72)	50.34
Former	1330	(22.08)	21.48
Current	1638	(27.20)	28.18
Alcohol consumption			
Never	1152	(19.13)	19.23
Moderate	3957	(65.70)	65.13
Severe	914	(15.18)	15.63
Physical activity			
No	3846	(63.86)	64.47
Low-intensity	1871	(31.06)	30.49
High-intensity	306	(5.08)	5.04
Occupational category			
Executive white-collar	1410	(23.41)	21.09
Ordinary white-collar	894	(14.84)	13.30
Pink-collar	1326	(22.02)	23.51
Green-collar	541	(8.98)	9.63
Skilled blue-collar	1030	(17.10)	17.54
Unskilled blue-collar	822	(13.65)	14.93
Work period			
Day	4942	(82.05)	81.88
Evening or night	654	(10.86)	11.10
Shift	427	(7.09)	7.02
Work status			
Paid worker	4093	(67.96)	65.07
Self-employed worker	1526	(25.34)	27.42
Unpaid family worker	404	(6.71)	7.51
Work duration, h/week			
< 43	2942	(48.85)	48.56
≥ 43	3081	(51.15)	51.44
Sunlight exposure, h/day			
< 2	3942	(65.45)	63.60
2–5	1325	(22.00)	22.94
≥ 5	756	(12.55)	13.47
Exposure to secondhand smoke at indoor workplace, h/day			
0	3228	(53.59)	52.60
0–1	2135	(35.45)	35.47
≥ 1	660	(10.96)	11.94

^a^Un-weighted %

^b^Weighted %

Based on the participants’ responses, 963 (16.0 %) individuals (men: 10.7 %; women: 21.6 %) reported symptoms of DES. Degree of obesity, smoking status, occupational category and employment type were the factors that were significantly associated with symptoms of DES. The DES group consisted of 344 men (34.8 %) and 619 women (65.2 %) (*P* < 0.0001). Distribution according to degree of obesity was 47.2 % of participants in the normal weight group, 23.7 % in the overweight group, and 29.0 % in the obese group, with the obese group having a significantly higher prevalence of symptoms of DES than the overweight group (*P* = 0.0008). Distribution according to smoking status was 60.6 % of participants in the nonsmoking group, 21.0 % in the current smoker group, and 18.38 % in the former smoker group, with current smokers having a higher prevalence of symptoms of DES than the former smokers. Distribution according to alcohol consumption was 69.0 % of participants in the moderate drinker group, 19.3 % in the non-drinker group, and 11.74 % in the heavy drinker group (*P* = 0.0035). The prevalence of symptoms of DES for occupational category was estimated 34.76 % in white-collar workers and 30.99 % in blue-collar workers. Distribution in DES group according to occupational status was 20.3 % of participants in the executive white-collar group, 17.4 % in the ordinary white-collar group, 24.8 % in the pink-collar group, 6.4 % in the green-collar group, 14.5 % in the skilled blue-collar group, and 16.6 % in the unskilled blue-collar group (*P* = 0.0002). Distribution according to work status was 70.6 % of participants in the paid work group, 21.7 % in the self-employed group, and 7.8 % in the unpaid group (Table 2).

**Table 2:** Association between variables and symptoms of dry eye syndrome

	DES group (*N* = 963)			Non-DES group (*N* = 5060)			
	No.	(%^a^)	%^b^	No.	(%^a^)	%^b^	*P*-value*
Sex							<0.0001
Men	344	(35.72)	34.79	2859	(56.50)	55.08	
Women	619	(64.28)	65.21	2201	(43.50)	44.92	
Age, years							0.1700
25–34	194	(20.51)	20.77	884	(17.76)	17.93	
35–44	250	(26.43)	24.17	1443	(28.99)	27.09	
45–54	281	(29.70)	30.71	1426	(28.65)	29.07	
55–65	221	(23.36)	24.35	1225	(24.61)	25.91	
Residence							0.4746
Urban	775	(80.48)	80.22	4049	(80.02)	78.60	
Rural	188	(19.52)	19.78	1011	(19.98)	21.40	
Household income							0.3838
1^st^ quartile	68	(7.06)	7.82	411	(8.12)	9.24	
2^nd^ quartile	246	(25.55)	27.62	1274	(25.18)	27.42	
3^rd^ quartile	289	(30.01)	30.30	1654	(32.69)	31.94	
4^th^ quartile	360	(37.38)	34.26	1721	(34.01)	31.40	
Education level							0.9961
Middle school	246	(25.55)	28.66	1306	(25.81)	28.75	
High school	352	(36.55)	37.39	1808	(35.73)	37.49	
University	365	(37.90)	33.95	1946	(38.46)	33.76	
Degree of obesity							0.0008
Normal	460	(47.77)	47.24	2026	(40.04)	40.07	
Overweight	222	(23.05)	23.73	1262	(24.94)	24.30	
Obese	281	(29.18)	29.03	1772	(35.02)	35.63	
Smoking							<0.0001
Never	600	(62.31)	60.66	2455	(48.52)	48.41	
Former	176	(18.28)	18.38	1154	(22.81)	22.06	
Current	187	(19.42)	20.96	1451	(28.68)	29.53	
Alcohol consumption							0.0035
Never	203	(21.08)	19.25	949	(18.75)	19.23	
Moderate	646	(67.08)	69.01	3311	(65.43)	64.41	
Severe	114	(11.84)	11.74	800	(15.81)	16.36	
Physical activity							0.3917
No	646	(67.08)	65.92	3200	(63.24)	64.20	
Low-intensity	278	(28.87)	29.94	1593	(31.48)	30.59	
High-intensity	39	(4.05)	4.14	267	(5.28)	5.20	
Occupational category							0.0002
Executive white-collar	222	(23.05)	20.33	1188	(23.48)	21.24	
Ordinary white-collar	170	(17.65)	17.39	724	(14.31)	12.53	
Pink-collar	221	(22.95)	24.80	1105	(21.84)	23.27	
Green-collar	68	(7.06)	6.36	473	(9.35)	10.24	
Skilled blue-collar	135	(14.02)	14.53	895	(17.69)	18.10	
Unskilled blue-collar	147	(15.26)	16.59	675	(13.34)	14.62	
Work period							0.2119
Day	783	(81.31)	82.08	4159	(82.19)	81.84	
Evening or night	121	(12.56)	12.20	533	(10.53)	10.90	
Shift	59	(6.13)	5.71	368	(7.27)	7.26	
Work duration, h/week							0.1519
< 43	496	(51.51)	51.32	2446	(48.34)	48.05	
≥ 43	467	(48.49)	48.68	2614	(51.66)	51.95	
Work status							0.0012
Paid worker	689	(71.55)	70.58	3404	(67.27)	64.04	
Self-employed worker	201	(20.87)	21.65	1325	(26.19)	28.50	
Unpaid family worker	73	(7.58)	7.76	331	(6.54)	7.46	
Sunlight exposure, h/day							0.1156
< 2	666	(69.16)	67.48	3276	(64.74)	62.87	
2–5	190	(19.73)	20.49	1135	(22.43)	23.39	
≥ 5	107	(11.11)	12.04	649	(12.83)	13.73	
Exposure to secondhand smoke at indoor workplace, h/day							0.7270
0	532	(55.24)	53.26	2696	(53.28)	52.47	
0–1	332	(34.48)	35.63	1803	(35.63)	35.44	
≥ 1	99	(10.28)	11.11	561	(11.09)	12.09	

*DES* dry eye syndrome

*Weighted *P*-value

^a^Un-weighted %

^b^Weighted %

Next, we analyzed the symptoms of DES incidence while considering multiple variables that are related to occupational characteristics and sex (Table 3).

**Table 3:** Multivariable analysis of gender-based relationship between occupational characteristics and symptoms of dry eye syndrome

	Model I^a^			Model II^b^		
	OR	95 % CI	*P*	OR	95 % CI	*P*
Occupational category						
Executive white-collar	1.40	(1.02–1.92)	0.035	1.36	(0.95–1.96)	0.096
Ordinary white-collar	1.73	(1.73–2.41)	0.001	1.70	(1.18–2.45)	0.005
Pink-collar	1.25	(0.92–1.69)	0.152	1.26	(0.90–1.75)	0.181
Green-collar	1.00			1.00		
Skilled blue-collar	1.44	(1.04–2.00)	0.028	1.48	(1.05–2.10)	0.027
Unskilled blue-collar	1.33	(0.97–1.82)	0.079	1.39	(1.00–1.95)	0.053
Work period						
Day	1.00			1.00		
Evening or night	1.08	(0.87–1.33)	0.507	1.07	(0.86–1.33)	0.555
Shift	0.99	(0.74–1.32)	0.933	1.00	(0.75–1.33)	0.974
Work duration, h/week						
< 43	1.00			1.00		
≥ 43	0.93	(0.81–1.07)	0.328	0.92	(0.80–1.07)	0.274
Work status						
Paid worker	1.21	(1.02–1.45)	0.033	1.22	(1.02–1.46)	0.029
Self-employed worker	1.00			1.00		
Unpaid family worker	0.95	(0.70–1.28)	0.728	0.96	(0.71–1.31)	0.802
Sunlight exposure h/day						
< 2	1.00			1.00		
2–5	0.95	(0.79–1.13)	0.536	0.96	(0.80–1.15)	0.679
≥ 5	1.01	(0.80–1.27)	0.943	1.06	(0.83–1.35)	0.631
Exposure to secondhand smoke at indoor workplace, h/day						
0	1.00			1.00		
0–1	0.91	(0.78–1.06)	0.221	0.90	(0.77–1.06)	0.199
≥ 1	0.99	(0.78–1.25)	0.899	0.97	(0.77–1.28)	0.822

*OR* odds ratio, *CI* confidence interval, *P p*-value

^a^Model I: Adjusted for age, sex and body mass index

^b^Model II: Adjusted for the variables in Model I, as well as socioeconomic status (education, household income and residence) and health behavioral factors (alcohol drinking, smoking and physical activity)

As the green-collar group had the lowest prevalence of symptoms of DES, it was used as the reference group. In Model I, the odd ratios (OR) for ordinary white-collar workers, executive white-collar workers and skilled blue-collar workers were significantly increased to 1.73 (95 % confidence interval [CI], 1.73–1.41), 1.40 (95 % CI, 1.02–1.92) and 1.44 (95 % CI, 1.04–2.00), respectively. Similar results were also obtained in Model II for ordinary white-collar workers (OR, 1.70; 95 % CI, 1.18–2.45), skilled blue-collar workers (OR, 1.48; 95 % CI, 1.05–2.10) and unskilled blue-collar workers (OR, 1.39; 95 % CI, 1.00–1.95).

When self-employed workers were used as the reference group, paid workers exhibited significantly higher ORs in Model I (OR, 1.21; 95 % CI, 1.02–1.45) and in Model II (OR, 1.22; 95 % CI, 1.02–1.46).

## Discussion

In the literature, various studies have reported the risk factors for DES, which include aging, medication, underlying pathological conditions and refractory surgery, although the association between DES and occupational conditions has not been adequately described. This study is the first one to examine the relationship between symptoms of DES and various occupational characteristics. To include only occupational factors as independent variables, we excluded unemployed participants and only included individuals who were 25–65 years old (the general working age in South Korea).

Based on the results of this study, white-collar workers had a higher risk of symptoms of DES than blue-collar workers (when using green-collar workers as the reference group). This finding allows us to conclude that symptoms of DES are influenced more by dry environments or occupations that involve intensive use of the eyes, such as prolonged work with documents or at a computer, rather than by exposure to microparticles or organic solvents. Our study was different from the previous studies. One study has also reported that working at a video display terminal increased the incidence of DES [25], although that study only evaluated office workers, and no comparison was made to other occupational categories. Furthermore, one study analyzed the occupational characteristics of DES, and reported that no significant results were found for the analysis categories [17], although these groups included unemployed subjects. Therefore, our findings are significant, as we have determined that office work is a more significant risk factor than manufacturing work.

Our finding that the green-collar workers had the lowest prevalence of symptoms of DES was different from our assumption, because we anticipated that outdoor works would lead to a higher prevalence of symptoms of DES, due to dust and ultraviolet radiation exposure. However, this finding is consistent with that of a previous study, which reported that the outdoor ambient humidity and environment had limited effects on the incidence of DES [26]. In addition, our assumption that age would be an important factor in the prevalence of symptoms of DES was also incorrect, likely because a high proportion of the older workers were employed in the Korean agriculture and fishing industries, and they exhibited a low prevalence of DES [27]. This finding is similar to the findings of a previous study that investigated the relationship between DES and Korean geographical characteristics [27]. Therefore, it appears that the prevalence of symptoms of DES are more closely related to an indoor work environment, rather than to the frequency of outdoor activities or age, among South Korean green-collar workers [28].

In the present study, sex, smoking status and occupational categories were significantly related to the prevalence of DES. Higher prevalence rates were observed among women (compared to among men), smokers (both current and former smokers), and among pink-collar workers (compared to green-collar workers). Interestingly, unskilled blue-collar workers exhibited a significantly higher incidence of symptoms of DES in Model II, although they also had a lower risk of symptoms of DES (relative to skilled blue-collar workers). This finding may be related to the Korean practice of promoting unskilled blue-collar workers to skilled blue-collar work, with a transition from manual work to white-collar work, such as video display terminal and document work, as they become more experienced and assume additional responsibility.

The finding that paid workers had a higher risk of symptoms of DES than self-employed workers can be attributed to the autonomy of self-employed workers to regulate their workplace environments. Similarly, the prevalence of symptoms of DES among paid white-collar workers is assumed to have been influenced by the degree to which they could control their workplace environments, which may explain why executive white-collar workers had a lower risk of symptoms of DES in Model I than ordinary white-collar workers. This explanation would indicate that higher autonomy in the workplace has a preventive effect on symptoms of DES, whereby white-collar work provides more workplace environmental autonomy (and a lower risk of symptoms of DES) than blue-collar work. However, this hypothesis requires validation in further studies. In addition, further studies are needed to confirm the higher risk of symptoms of DES for paid workers (compared to self-employed workers), as other factors, such as work intensity and work-related stresses, may also affect the incidence of symptoms of DES.

This study’s findings demonstrate that indoor workers have a higher prevalence of DES than workers who are employed in agricultural work. A particularly high risk of DES was noted for white-collar workers, which is consistent with the findings of previous studies. However, to our knowledge, this study is the first to reveal that white-collar workers had a higher risk of DES than blue-collar workers, although this aspect requires further investigation in future studies. An important implication of these findings is that the change in the incidence of DES may be related to changes in work environment that are associated with hierarchical changes among skilled and unskilled blue-collar workers. Furthermore, in addition to the established risk factors for DES (old age, female sex, smoking, use of contact lenses, refractory surgery and dry environment), this study revealed that ordinary white-collar, skilled blue-collar and unskilled blue-collar workers were at an increased risk of DES. Therefore, although the study design precludes the direct application of the occupation-related risk of DES in determining an individual’s risk of DES (given the differences among the occupational categories), it is advisable to seriously consider these occupation-related risk factors when a patient is clinically diagnosed with DES. Finally, the fact that paid workers had a significantly higher risk of DES, compared to self-employed workers, is a novel finding. Thus, the results of this study are expected to arouse a keen interest in DES-related occupational factors, and to motivate continued research efforts to evaluate the relationship between DES and occupational characteristics.

The strength of this study is that its findings are based on an authoritative nationwide database. By using data from the recent KNHANES, the results of our analysis can be considered representative of the national Korean status and tendencies. Furthermore, our sample size (*n* = 6023) exceeds that of the largest previous study of DES [13]. Finally, the associations between multiple occupational characteristics and the risk of DES were established, which has not been observed in previous studies, due to the lack of controlling for non-occupational variables. Therefore, this study’s findings are significant, as they present the first statistically significant conclusions regarding symptoms of DES-related occupational risk factors.

One limitation of this study is that the symptoms of DES were based on a self-reported questionnaire. This study applied a self-reported questionnaire for DES diagnosis, because the use of a self-reported questionnaire is common in DES-related studies and its reliability has been clinically verified [21, 29]. Moreover, to estimate the symptoms of DES which has dryness and irritations that are the most reliable symptoms to diagnose DES. [30–32]. Clinically, medications to mitigate clinical symptoms are generally administered based on patient complaints [33]. For this reason, the use of self-reported questionnaire is thought to be one of the most reliable methods to diagnose DES. Another limitation of this study was its cross-sectional design, which precludes us from determining the causal direction of the relationship between DES and occupational characteristics.

## Conclusions

This study tries to find the risk of symptoms of DES from the perspective of occupational category. This is the first study to reveal that white-collar workers have a higher risk of symptoms of DES than blue-collar workers, that skilled blue-collar workers have a higher risk than unskilled blue-collar workers, and that paid workers have a higher risk than self-employed workers, that shows autonomy at the workplace is also important factor to estimate the risk of symptoms of DES.

## Abbreviations

DES: Dry Eye Syndrome
NHANES: The Korean National Health and Nutrition Examination Survey
KCDC: The Korean Centers for Disease Control and Prevention
BMI: Body mass index
OR: Odds ratio
IRB: Institutional review board
